# Chemically defined, ultrasoft PDMS elastomers with selectable elasticity for mechanobiology

**DOI:** 10.1371/journal.pone.0195180

**Published:** 2018-04-06

**Authors:** Viktor Heinrichs, Sabine Dieluweit, Jörg Stellbrink, Wim Pyckhout-Hintzen, Nils Hersch, Dieter Richter, Rudolf Merkel

**Affiliations:** 1 Institute of Complex Systems 7, Forschungszentrum Jülich GmbH, Jülich, Germany; 2 Juelich Centre for Neutron Science 1 and Institute of Complex Systems 1, Forschungszentrum Jülich GmbH, Jülich, Germany; Institute of Materials Science, GERMANY

## Abstract

Living animal cells are strongly influenced by the mechanical properties of their environment. To model physiological conditions ultrasoft cell culture substrates, in some instances with elasticity (Young's modulus) of only 1 kPa, are mandatory. Due to their long shelf life PDMS-based elastomers are a popular choice. However, uncertainty about additives in commercial formulations and difficulties to reach very soft materials limit their use. Here, we produced silicone elastomers from few, chemically defined and commercially available substances. Elastomers exhibited elasticities in the range from 1 kPa to 55 kPa. In detail, a high molecular weight (155 kg/mol), vinyl-terminated linear silicone was crosslinked with a multifunctional (f = 51) crosslinker (a copolymer of dimethyl siloxane and hydrosilane) by a platinum catalyst. The following different strategies towards ultrasoft materials were explored: sparse crosslinking, swelling with inert silicone polymers, and, finally, deliberate introduction of dangling ends into the network (inhibition). Rheological experiments with very low frequencies led to precise viscoelastic characterizations. All strategies enabled tuning of stiffness with the lowest stiffness of ~1 kPa reached by inhibition. This system was also most practical to use. Biocompatibility of materials was tested using primary cortical neurons from rats. Even after several days of cultivation no adverse effects were found.

## Introduction

Reaction to external signals is one of the hallmarks of living systems. For many years, research on living cells has focused on chemical and genetic factors. However, recently mechanical factors have emerged as additional potent control parameters [1, 2]. For example, many animal cells strongly change their phenotypes in response to the elasticity of their substrates. Moreover, cells have been shown to generate mechanical forces and to react to them [3, 4]. Such mechanobiological effects are well documented, amongst others, for endothelial cells, smooth muscle cells, stem cells, and fibroblasts [5–9].

These findings have far reaching consequences. For example, to obtain results relevant for physiology *in vitro* studies on cells should be done under nature-like mechanical conditions. Conversely, tailoring mechanical conditions during cell cultivation is currently investigated as route towards fast and efficient growth of engineered tissues for medical applications [10, 11].

Obviously, cell culture substrates with close to nature mechanical properties are of highest interest for mechanobiology [12–14]. However, extracellular matrix materials are not only extremely soft but they also exhibit time-dependent mechanical response, i.e. they are viscoelastic. Therefore, the mechanical resistance experienced by cells is time dependent. For example, heart muscle cells change their forces within milliseconds, while during migration of breast cancer cells forces persist for hours. Therefore, these cells will sense very different stiffness for the same viscoelastic substrate. Thus, an in-depth analysis of a potential cell culture substrate should also include measurements on viscoelastic response.

The challenge is to mimic tissue, here discussed for brain tissue whose mechanical properties were reviewed by Chatelin et al. [15]. Absolute values of shear modulus reported varied substantially, mostly in the range from 0.1 kPa to 3 kPa [16, 17], but all measurements show an increase of shear modulus G with increasing frequency. As most techniques used rely on shearing small tissue samples, this modulus is customarily reported. It can be converted to Young's modulus E that is usually given in *in vitro* cell culture work by E = 2G(1+*v*). Poisson's ratio, *v*, is a dimensionless number between 0 and 0.5. As most ultrasoft materials (including the ones described in this article) deform at constant volume, their Poisson's ratio is 0.5 and the Young's modulus is simply three times the shear modulus.

Indeed, ultrasoft hydrogels with Young's modulus of 1 kPa were already accomplished on polyacrylamide basis [1]. However, these materials cannot be stored for a long time after preparation and they show little frequency dependence of the mechanical response. As alternative, silicone elastomers have been used as soft substrates for cell culture [3, 18]. Indeed, such elastomers can be used to mimic brain tissue mechanics (head models) in automotive crash and ballistic tests [19]. There are already some rheological studies on PDMS elastomers [18, 20]. However, an ideal cell culture substrate from silicone should be prepared from few, well characterized and easily accessible chemicals, should be non-toxic, suitable for light microscopy, and its stiffness should be tunable down to the ultrasoft regime of about 1 kPa. To our knowledge there are few such systems and no systematic rheological studies on any of them. Here, we set out to fill this gap.

For a knowledge-based approach towards ultrasoft materials we relied on rubber theory [21–23]. This theory describes the mechanical response of fully crosslinked elastomers with the major outcome

$$G\propto \rho k_{B}T$$ (1)

where ρ is the number density of elastically effective crosslinks per unit volume, k_B_ Boltzmann's constant and T absolute temperature. The proportionality factor is on the order of one. For ideal networks the crosslink density is given by the mixing ratio of the two network constituents, bifunctional linear chain and multifunctional crosslinker. So, in principle, the elastic properties of the network can be adjusted via composition, i.e., by varying the crosslinker stoichiometric ratio r defined as:

$$r=\frac{f_{cl}n_{cl}}{f_{p}n_{p}}$$ (2)

Here, f denotes the functionality of the molecule, n its molar amount and the indices cl and p indicate crosslinker and polymer, respectively. Theoretically, the shear modulus G reaches a maximum G at r = 1 (stoichiometrically balanced networks). But for real networks the maximum of the shear modulus occurs at higher stoichiometric ratio (r > 1.3) [24, 25].

In summary, each parameter that reduces the density of elastically active crosslinks may be used to reach ultrasoft systems. The most important factor is certainly the molecular weight of the bifunctional linear chain because its volume defines an upper limit for the crosslink density. But also reaction kinetics (by changing the catalyst concentration) [26–28], amount of the sol fraction (by adding an inert polymer that swells the network and thus separates crosslinks) [29], or network defects (by inhibiting some functional groups of the crosslinker) [18] have a crucial influence on network elasticity.

Here, we compared these different strategies. We present rheological results from stoichiometrically balanced and unbalanced silicone (PDMS) elastomers proposed as elastically well-defined and at the same time adjustable substrates in mechanobiology. The following strategies towards ultrasoft elastomers were tested: i) reduction of the catalyst concentration, ii) variation of the crosslinker stoichiometric ratio r (system 1 –neat networks), iii) addition of inert filler polymers of different molecular weight (system 2 –swollen networks), and, finally, iv) partial blocking of functional groups of the crosslinker (system 3 –inhibited networks).

## Materials and methods

### Sample preparation

Unless mentioned otherwise, all chemicals were purchased from ABCR GmbH, Karlsruhe, Germany and used without further purification.

#### System 1: Neat PDMS networks

Neat networks were formed from polydimethyl-siloxane (PDMS) using end-terminated divinyl-polydimethyl-siloxane (DMS-V52, M_n_ = 155 kg/mol, f_p_ = 2) as bifunctional linear network chain and a copolymer on hydrosilane base as multi-functional crosslinker (HMS-064, M_n_ = 55–65 kg/mol, f_cl_ = 51). All PDMS networks were characterized by their stoichiometric ratios r, defined as the ratio of the functional groups hydrosilane to vinyl groups (see Eq 2). The hydrosilylation reaction was catalyzed by a platinum complex (Karstedt catalyst SIP 6830.3). An overall catalyst concentration in the range from 0.5 to 1 ppm gave optimal results; to facilitate mixing the catalyst was diluted in divinyl-polydimethyl-siloxane (0.1%).

The components were thoroughly mixed manually and degassed. Subsequently, the mixtures were deposited carefully between two parallel plates (stainless steel, diameter 25 mm, 1 mm gap) of a strain controlled rheometer (ARES-G2, TA Instruments, New Castle, Delaware, USA). All samples were cured directly in the instrument for at least 15 hours at room temperature.

#### System 2: Swollen PDMS networks

These were prepared as follows. In the beginning, i.e. before adding the catalyst, linear trimethylterminated polydimethylsiloxane (DMS-T31, M_n_ = 28 kg/mol, DMS-T41.2, M_n_ = 68 kg/mol, or DMS-T51, M_n_ = 139 kg/mol) was additionally mixed to the precursors (system 1). Further treatment was done as above.

#### System 3: Inhibited PDMS networks

Inhibited networks were prepared using a chemical agent competing for active crosslink sites. Monovinyl-terminated alkane (10-undecenyl 2-bromoisobutyrate, M_n_ = 319 g/mol, Sigma-Aldrich, St. Louis, Missouri, USA) was added to the educts (system 1). As this chemical reduces the activity of the catalyst, curing had to be done at elevated temperature (80°C).

### Determination of the sol fraction

The sol fraction, i.e. the amount of material not covalently linked to the continuous elastomer network, was determined by solvent extraction. Four samples (0.5–1 g) of each ratio (r = 1.28, 0.84, 0.71, swollen 0.71) were immersed in cyclohexane (ca. 50 mL) and shaken for 24 hours. During this time the sample volume increased tenfold and free molecules diffused into the surrounding cyclohexane phase that was exchanged several times to maintain a concentration gradient. Cyclohexane was collected and evaporated. The washed samples were dried. Afterwards both portions were weighted. No further experiments were done on these samples because they were of irregular shape and could be easily damaged.

### Rheology

Dynamic oscillatory shear measurements were performed with a strain-controlled rheometer, (ARES-G2, TA Instruments, New Castle, Delaware, USA) in the linear viscoelastic region. Three different measurement protocols were used: a) amplitude sweeps were performed at room temperature (20°C) at fixed angular frequencies of 0.628, 6.28, and 62.8 rad/s, respectively, in an amplitude range from 0.01 to 100% strain; b) frequency sweeps were performed at 1% strain and room temperature or 37°C in an angular frequency range from 10^−5^ rad/s to 100 rad/s; and c) time resolved sweeps were done at fixed frequency of 6.28 rad/s, 1% strain mainly at room temperature; for selected samples also at 80°C. These time sweeps lasted up to several days depending on reaction kinetics and therefore intermittent short frequency sweeps, 10^−2^ to 100 rad/s, were performed for further monitoring network formation. Following time sweeps, the final elastomer was characterized by amplitude and frequency sweeps.

Temperature stability within ±0.5°C was achieved by a Peltier element. Time-temperature superposition was not used, that is, all data were directly acquired by the rheometer. Reproducibility was within 10%.

### Experiments on cells

Cortical neurons were isolated from 19-day-old Wistar rat embryos as described in [30]. These experiments were approved by the local ethics committee (animal testing permit No. 84–02.04.2015.A173; Landesamt für Natur, Umwelt und Verbraucherschutz Nordrhein-Westfahlen). Pregnant rats were CO_2_ anesthetized and subsequently decapitated. All efforts were made to minimize suffering. Cells were collected by centrifugation at 200 g, maintained at 37°C and 5% CO_2_ in a humidified incubator and cultured in neurobasal medium supplemented with 1% B27, 0.5 mM L-glutamine and 50 μg/ml gentamycine (Sigma-Aldrich, Germany). Neurons were seeded on poly-L-lysine coated (Sigma-Aldrich, Germany) (1%, 30 min at 37°C) PDMS substrates (r = 0.71, system 1 and r = 3.18, system 3) and cultivated for at least 5 days. Live cell analyses were performed at 37°C and 5% CO_2_ (cell incubator KL2, Zeiss, Germany) using a confocal laser scanning microscope (LSM 710, Zeiss, Germany) using a 20x Plan-Apochromat (Ph2, NA 0.8, Zeiss, Germany). Phase contrast micrographs were taken using an argon ion laser (488 nm).

## Results

### Establishment of the basic system

**Fig 1:**
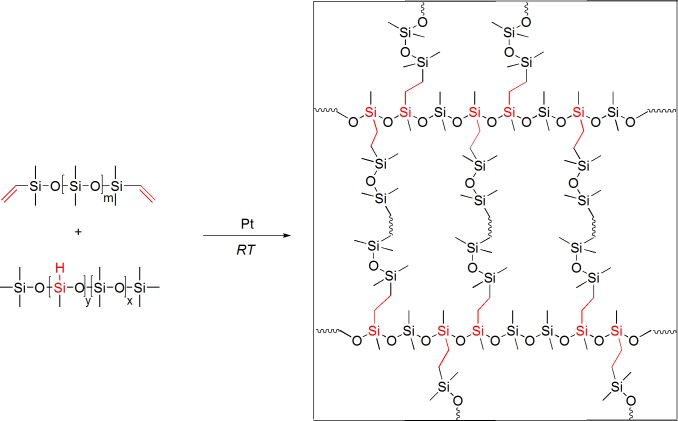
Schematic diagram of the PDMS system for preparation of ultrasoft PDMS elastomers; m ≈ 2090; x ≈ 700, y ≈ 51, calculated from 5–7 mol% of hydrosilane group. Right hand side shows a detail of the PDMS network.

Our primary aim was to develop and optimize an ultrasoft elastomeric material suitable for a broad range of cell biological experiments. Specifically, we aimed at a system prepared from few and well-characterized basic materials and a simple and reliable protocol. To achieve this, polydimethylsiloxane (PDMS) was crosslinked by hydrosilation using a Karstedt catalyst (cf. Fig 1). Because low stiffness requires a low spatial density of active crosslinks (cf. Eq 1), high molecular weight compounds were chosen. In detail, end-terminated divinyl-PDMS (M_n_ = 155 kg/mol) was utilized as linear network strands and a statistical copolymer (M_n_ = 55–65 kg/mol) with dimethyl- and methylhydro-siloxane units as functional groups (functionality f_cl_ = 51) served as multi-functional crosslinker.

Neat and swollen PDMS networks were produced at room temperature directly in the rheometer. Progress of the reaction was followed by time sweeps and the success of curing confirmed by the crossing of G'(ω) and G"(ω).

Once curing was complete, oscillatory shear rheology was used to quantify viscoelastic behavior of networks. All materials acted as viscoelastic solids, therefore exact knowledge of the frequency dependence of storage and loss modulus, i.e., G'(ω) and G"(ω), was mandatory. Furthermore, the equilibrium shear modulus, i.e., the low frequency limit of G'(ω), can be converted into Young's modulus E. Neat networks (system 1) with stoichiometric ratios, r, of 0.71, 0.84, 0.92, 1.00, 1.14, 1.21, and 1.28, respectively, were examined. For the definition of r see Eq 2.

**Fig 2:**
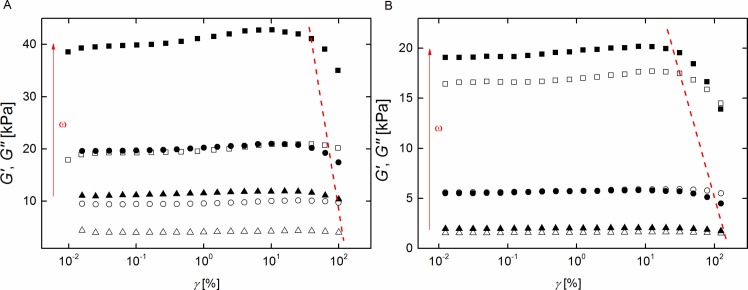
Silicone elastomers exhibit a large linear range. Amplitude sweeps for neat silicone elastomers (system 1) with r = 1 (A) and r = 0.71 (B) measured at 20°C. Storage (solid symbols) and loss (open symbols) modulus are plotted against strain amplitude. Squares ω = 62.83 rad/s; circles ω = 6.283 rad/s; and triangles ω = 0.6283 rad/s. The dashed lines indicate the limits of the linear regime. Raw data can be found in S1 Dataset.

First, oscillatory rheology measurements were performed with moderately varying deformation amplitude at fixed frequency (strain sweeps) to determine the linear viscoelastic regime of each sample. Herein, the strain amplitude γ was increased from 0.01% to 100% at fixed frequencies ω of 0.63 rad/s, 6.28 rad/s, and 62.8 rad/s, respectively. Fig 2 shows typical results.

Below a certain threshold, storage and loss modules G' and G" were independent of amplitude. This linear regime extended to very high strains, especially for low frequencies. All subsequent measurements were performed at a strain amplitude of 1%, well in the linear regime.

### System 1 –neat networks: Varying the catalyst concentration

In principle, one possibility to decrease the equilibrium shear modulus G_0_ is to reduce the catalyst concentration. Due to kinetic effects, a slower curing reaction leads to larger molecular weights between crosslinks and thus to lower stiffness (cf. Eq 1). Moreover, reduction of the catalyst concentration results in longer work time, for details see Supporting Information (S1 Fig, S2 Dataset and S1 Table).

**Fig 3:**
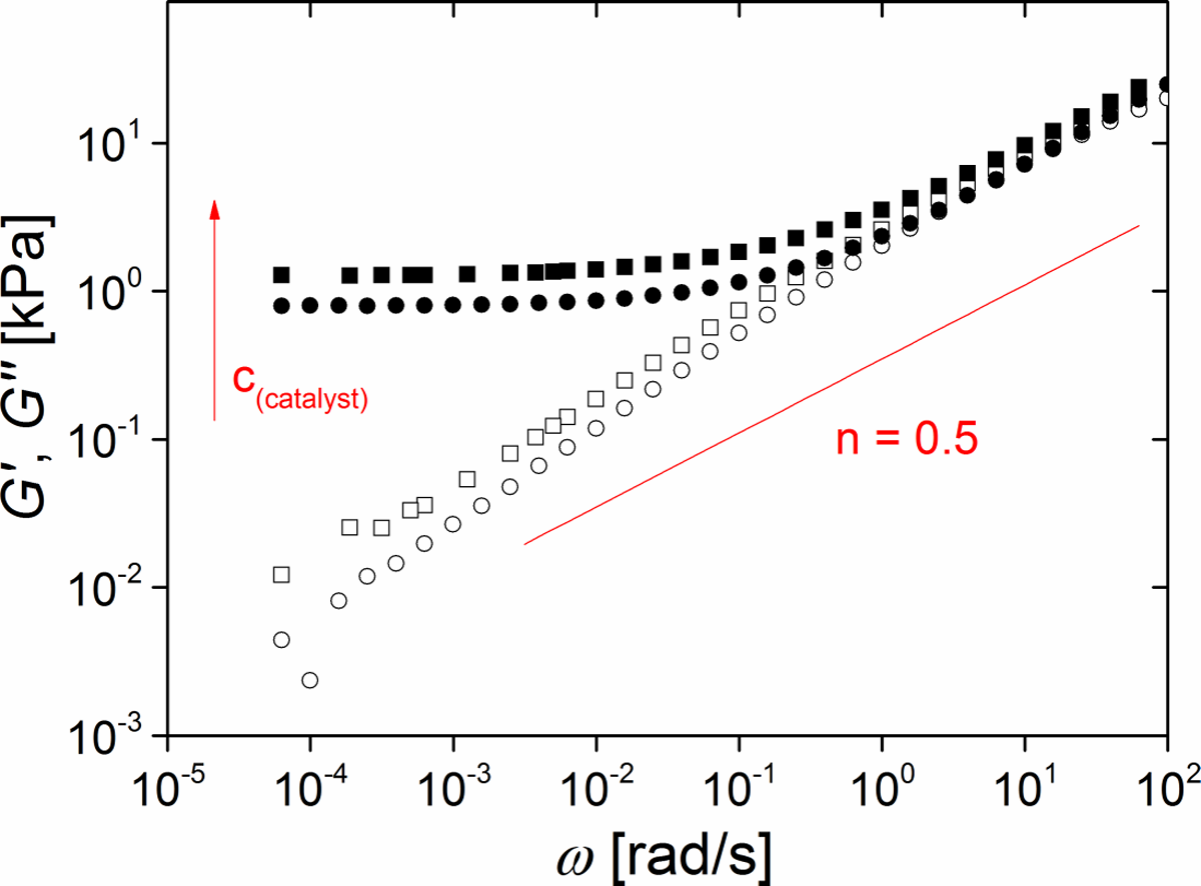
Reducing the catalyst concentration decreases elasticity of elastomers. Frequency dependence of storage (G', solid symbols) and loss modules (G", open symbols) of neat silicone networks (system 1) prepared at r = 0.71 and measured at 1% strain. Squares: 0.5 ppm catalyst; circles: 0.37 ppm catalyst. Red line: power law expected for a critical gel (see discussion). Raw data can be found in S3 Dataset.

Our experiments clearly showed that reducing the catalyst concentration from 0.5 ppm to 0.37 ppm results in softer networks. A typical result is shown in Fig 3. Please note, for both catalyst concentrations equilibrium shear stiffness, indicated by constant values of the storage modulus G', is reached at angular frequencies below 10^−2^ rad/s. Moreover, for both concentrations loss modules, G'', follow a power law ω^n^ with n≈0.6 over a wide range of frequencies (from 10^−4^ rad/s to 1 rad/s). This is a clear indication for a broad distribution of frequencies where stress relaxation takes place [31]. At higher frequencies both modules approach each other and follow approximately the same power law with n ≈ 0.5 as expected for critical gels [31]. We interpret the slight bending down observed for the lower catalyst concentration at highest frequencies as onset of the rubber plateau that results from entanglements of linear polymers [21–23].

In summary, diminishing the catalyst concentration by approximately 25% yielded a 33% reduction of the equilibrium shear modulus G_0_, from 1.2 kPa to 0.8 kPa, for a stoichiometric ratio r = 0.71. We found the same effect for r = 1.28 where we found 18.3 kPa at 0.5 ppm catalyst and 12.4 kPa at 0.37 ppm. However, further reduction of the catalyst concentration down to 0.11 ppm resulted in poor reproducibility and incomplete curing. For the sake of reliability and error tolerance we performed all following experiments at a catalyst concentration of 0.5 ppm.

### System 1 –neat networks: Varying the stoichiometric ratio

**Fig 4:**
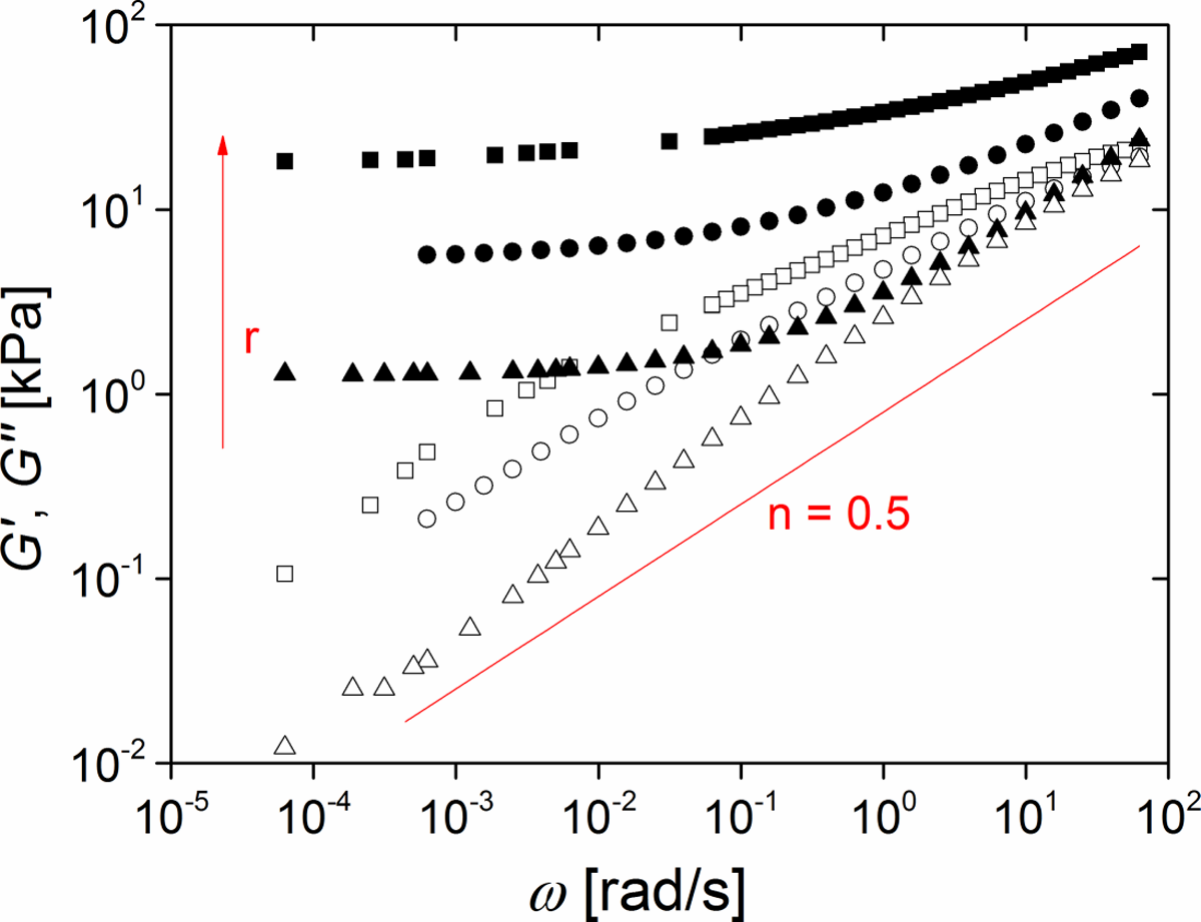
The stoichiometric ratio controls viscoelastic response. Frequency dependence of storage (G', solid symbols) and loss modules (G", open symbols); squares stoichiometric ratio r = 1.28; circles: r = 1.00; triangles: r = 0.71. Red line: power law expected for a critical gel (see discussion). Raw data can be found in S4 Dataset.

Fig 4 shows typical frequency sweep data of PDMS elastomers with different stoichiometric ratios r. All samples (r = 0.71, 0.84, 0.92, 1.00, 1.14, 1.21, and 1.28, respectively) displayed a clear plateau of the storage modulus G' at low frequencies.

Indeed, curing samples of stoichiometric ratio as small as r = 0.58 yielded unreliable results. While some samples formed gels with G_0_ ≈ 0.5 kPa, in most cases only fully viscous behavior was found. So G_0_ = 1.25 kPa obtained for r = 0.71 appears to be the minimum elasticity that can be reliably achieved by variation of the stoichiometric ratio alone. Moreover, the storage modulus G' dominated the loss modulus G", i.e. G' > G". Therefore all samples were viscoelastic solids at all frequencies.

The equilibrium shear modulus, G_0_, was approximated by the storage modulus measured at the lowest angular frequency used (typically 6.28 10^−4^ rad/s or 6.28 10^−5^ rad/s). This simple procedure required lengthy measurements at extremely low angular frequencies but avoided all problems connected with data fitting and extrapolation. The equilibrium shear modulus decreases with decreasing stoichiometric ratio by one order of magnitude and reaches the ultrasoft regime with G_0_ ≈ 1 kPa, see Table 1.

**Table 1:** Dependence of the equilibrium shear modulus G_0_ on the stoichiometric ratio r.

r	1.28	1.21	1.14	1.0	0.92	0.84	071
G_0_ [kPa]	18.3	16.5	13.3	5.7	4.5	3.8	1.2

Although both modules G' and G" increased and approached each other at higher frequency, no crossover was observed in the experimental frequency range (cf. Fig 4). With decreasing stoichiometric ratio r this approach became very close. The balance between G' and G" is usually quantified by the dissipation factor, tan(δ), defined as quotient of G" and G'. A value of 0 indicates fully elastic behavior and ∞ fully viscous one. Values of the dissipation factor at a high and a low frequency are shown in Table 2. Obviously, dissipation increases with lower crosslinking (lower r) and becomes almost negligible at very low angular frequency, which again indicates elastic behavior at long time scales.

**Table 2:** Dissipation factors of neat silicone networks.

	tan (δ)	
r	ω = 10 rad/s	ω = 6.3 10^−3^ rad/s
1.28	0.288	0.066
1.00	0.492	0.098
0.71	0.817	0.080

Dissipation factors, tan(δ) = G''/G', for different stoichiometric ratios and two different angular frequencies.

**Fig 5:**
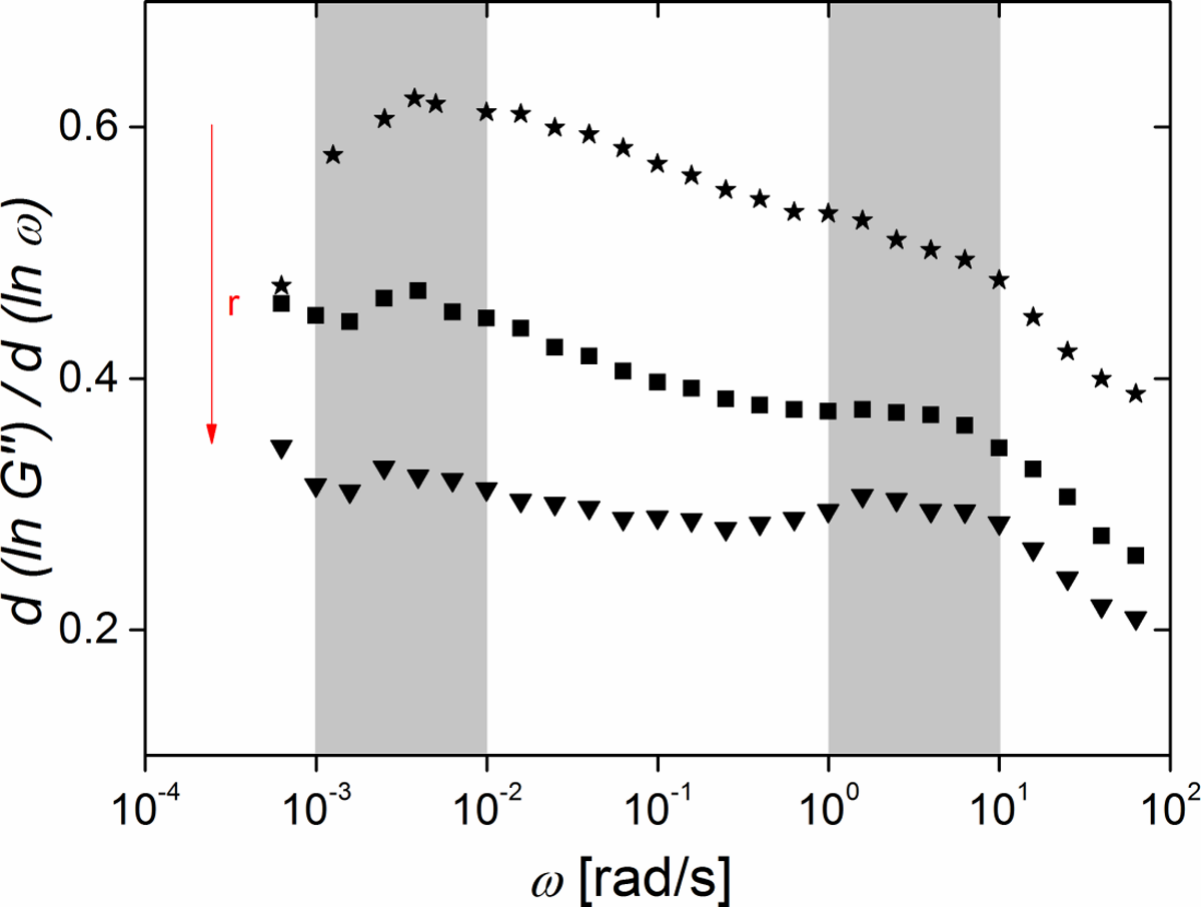
The loss module may be approximated by power laws in some regions. The local slope of G'' in a double logarithmic plot, i.e. the effective exponent n, as a function of angular frequency; (triangles) elastomer with r = 1.21; (squares) r = 0.84; (stars) r = 0.71. Raw data can be found in S5 Dataset.

As can be seen from Fig 4, the detailed frequency dependence of G' and G" is rather complex but at least for the loss module G'' some approximate power law regimes can be identified. This can be directly seen in Fig 5 where the local power law exponent n, given by the slope of ln G'' as function of ln ω, is shown.

The local power law exponents clearly varied with frequency and stoichiometric ratio. However, all samples showed a marked decrease of n at very high frequencies. This decay originates from the approach to the rubber plateau, which results from entanglements of network strands [21–23, 32].

As we were investigating networks with very low crosslink densities, we expected the infinite network (the gel) to contain a certain amount of silicone polymers not covalently connected to the network (the sol). This was quantified by solvent extraction (cf. Materials and Methods). The results (cf. Table 3) show a pronounced increase of the sol fraction with decreasing stoichiometric ratio r.

**Table 3:** Dependence of sol fraction on stoichiometric ratio.

r	1.28	0.84	0.71	0.71^a^^)^
added inert PDMS [wt%]	0	0	0	25
w_Sol_ [wt%] ± s.d.	35±14	53±4	73±12	78±12

a) system 2

Sol fraction of PDMS-elastomers obtained by solvent extraction. Here, r symbolizes stoichiometric ratio, w_Sol_ the weight fraction of sol. Each experiment was repeated four times. Raw data are shown in S6 Dataset.

The sol fraction could be increased by addition of an inert filler polymer, system 2, as will be discussed in detail in the following section. The sol fraction of this sample is consistent with expectations from simple volumetric calculations.

Please note that the sol fraction did not change with age of the sample. Due to the chosen high molecular weights of all components, release of sol fraction without swelling was absent as indicated by long time rheological (3 days) as well as cell culture experiments (5 days, see below).

### System 2—swollen networks: Adding inert filler polymers

As mentioned in the Introduction, increasing the sol fraction by adding a low molecular weight solvent leads to increasing distances between crosslinking and thus further reduction of G_0_ (cf. Eq 1). Here, we purposely increased the sol fraction by addition of a chemical inert, trimethyl-terminated PDMS polymer, namely before curing.

The choice of the molecular weight of the polymer added was a compromise. On one hand Mrozek et al. showed that elasticity decreases with increasing amounts of inert polymer added and decreasing molecular weights of them [25]. However, at low frequencies the latter effect is only marginal. On the other hand, separation from the network is prevented by large molecular weight, exceeding the critical molecular weight of PDMS, M_c_ = 2M_e_ ≈ 29 kg/mol [18]. Therefore, we used an inert polymer with a molecular weight M_n_ = 28 kg/mol, slightly below M_c_.

**Fig 6:**
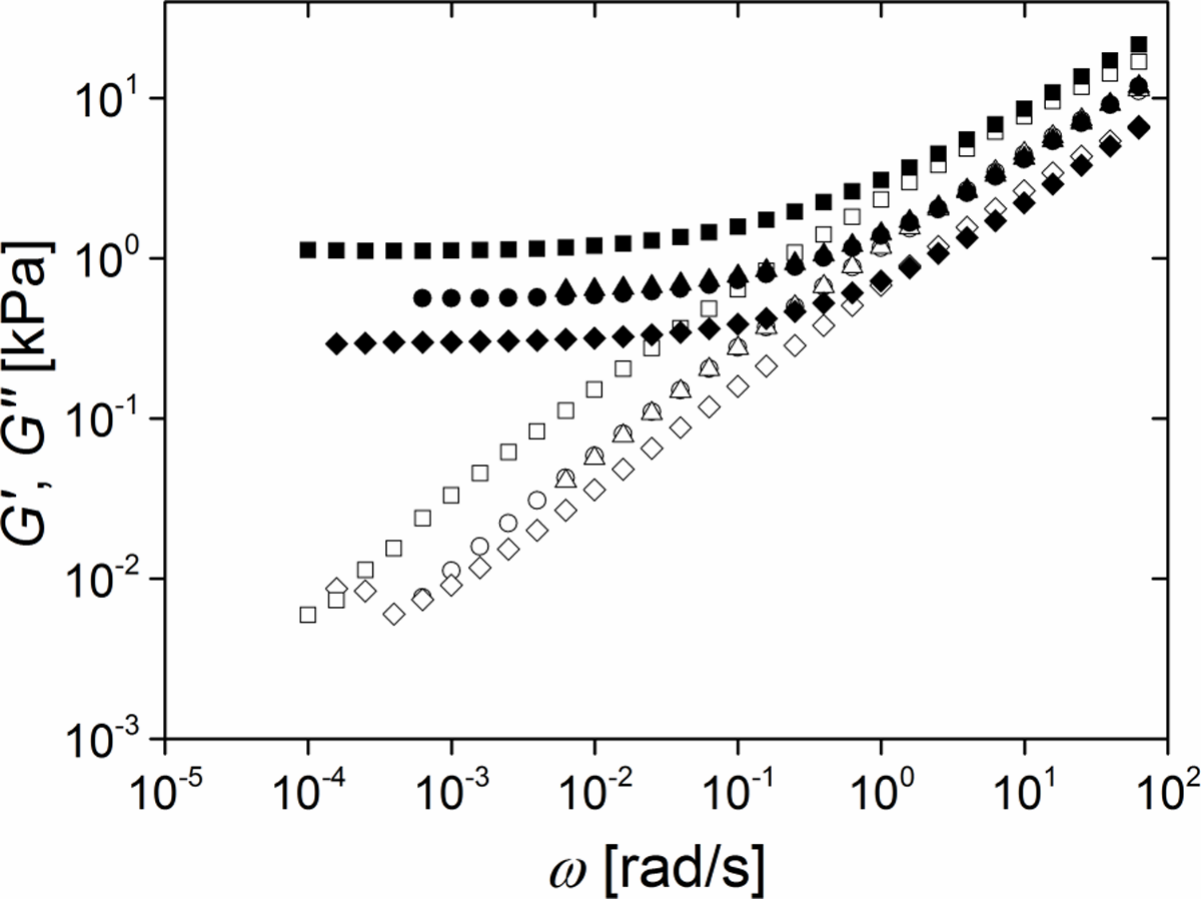
Change of rheological properties due to addition of inert filler polymers. Frequency dependence of the storage (G', solid symbols) and loss modules (G", open symbols) at a strain of 1%. All samples: r = 0.71 and 0.5 ppm catalyst; Squares: neat system; Circles: 25% (v/v) of 139 kg/mol inert PDMS added; Triangles: 25% (v/v) of 68 kg/mol inert PDMS added; Diamonds: 25% (v/v) of 28 kg/mol inert PDMS added. Raw data can be found in S7 Dataset.

Fig 6 shows the mechanical effects of the addition of 25% (v/v) of this inert polymer at otherwise identical parameters. Clearly, storage (G') and loss (G") modulus were decreased over the whole frequency range. The equilibrium shear modulus reached G_0_ ≈ 0.3 kPa, i.e. it was reduced by approximately 75%. Increase of the molecular weight of the inert polymer to 68 kg/mol or 139 kg/mol—while keeping its volume fraction Φ constant at 25%—yielded only a reduction of the equilibrium shear modulus to G_0_ ≈ 0.6 kPa. This was most likely due to the fact that both molecular weights were already well above the critical molecular weight.

For all swollen networks the differences between G' and G'' became marginal at higher frequencies ω ≥ 3 rad/s. For the lowest molecular weight of the inert polymer, M_n_ = 28 kg/mol, even a crossing of storage G' and loss modulus G" was observed. That is, for this system viscous contributions dominated at high frequencies which could complicate the interpretation of mechanobiological experiments.

### System 3—inhibited networks: Varying the amount of dangling ends

Another strategy toward softer elastomers is to intentionally increase the number of network defects, so called dangling ends acting as internal plasticizers. These are polymer strands that are only at one end covalently connected to the network and therefore cannot contribute to its elasticity. Here, this was achieved by adding monovinyl-functionalized chains that competed with the divinyl-functionalized chains during the curing reaction. We choose low molecular weight monovinyl alkane ester for this purpose, in the following also called inhibitor.

**Fig 7:**
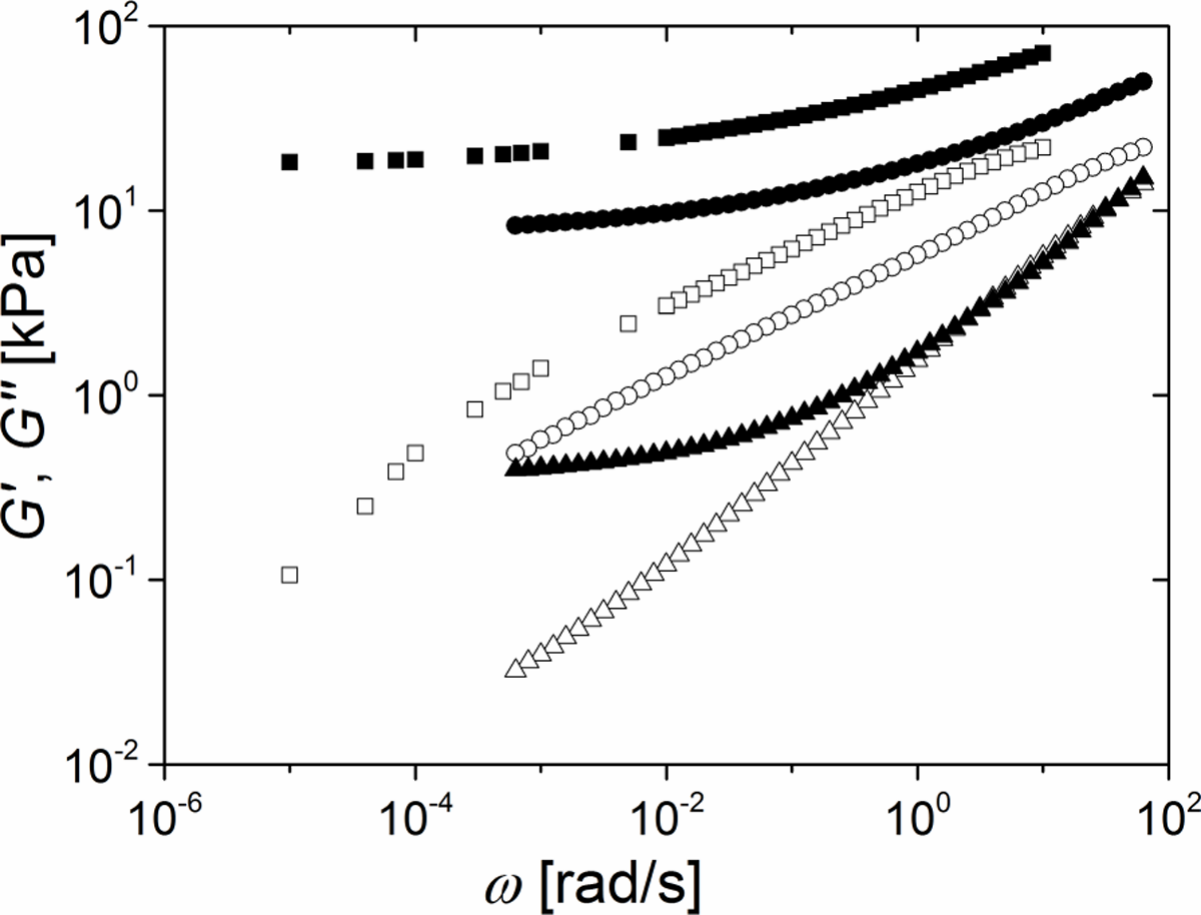
Inhibition results in ultrasoft elastomers. Frequency dependence of the storage (G', solid symbols) and loss modules (G", open symbols). All measurements 1% strain and stoichiometric ratio 1.28. Squares: 0.5 ppm catalyst and c_inhibitor_ = 0% (neat system); Circles: 0.5 ppm catalyst and w_inhibitor_ = 0.25%; Triangles: 1.0 ppm catalyst and c_inhibitor_ = 2%. Raw data can be found in S8 Dataset.

Since the monovinyl alkane ester inhibited the catalyst, higher amounts of catalyst, see below, and higher curing temperatures, 80°C instead of 20°C, were necessary to initiate elastomer formation. Moreover, curing started only after temperature was increased. Therefore, compared to systems 1 and 2, this system enables much longer work time and, thus, much easier handling. With this system a catalyst concentration of 1 ppm gave stable and reliable results. As can be seen in Fig 7, system 3 results in much softer elastomers than could be achieved with non-inhibited networks. For example, even at a rather high stoichiometric value of 1.28, which gave G_0_ = 18.3 kPa as a neat network, addition of a small amount, 0.25%, of inhibitor reduced the shear stiffness by more than 50% to G_0_ = 8.3 kPa.

Further increase of the amount of inhibitor to 2% resulted in the production of one of the softest elastomers achieved in this study. This material showed an equilibrium shear modulus as low as G_0_ = 0.4 kPa well in the ultrasoft regime. As for all frequencies tested the storage module, G', is larger than the loss module, G'', the mechanical behavior of these elastomers was mostly elastic.

### Biocompatibility

**Fig 8:**
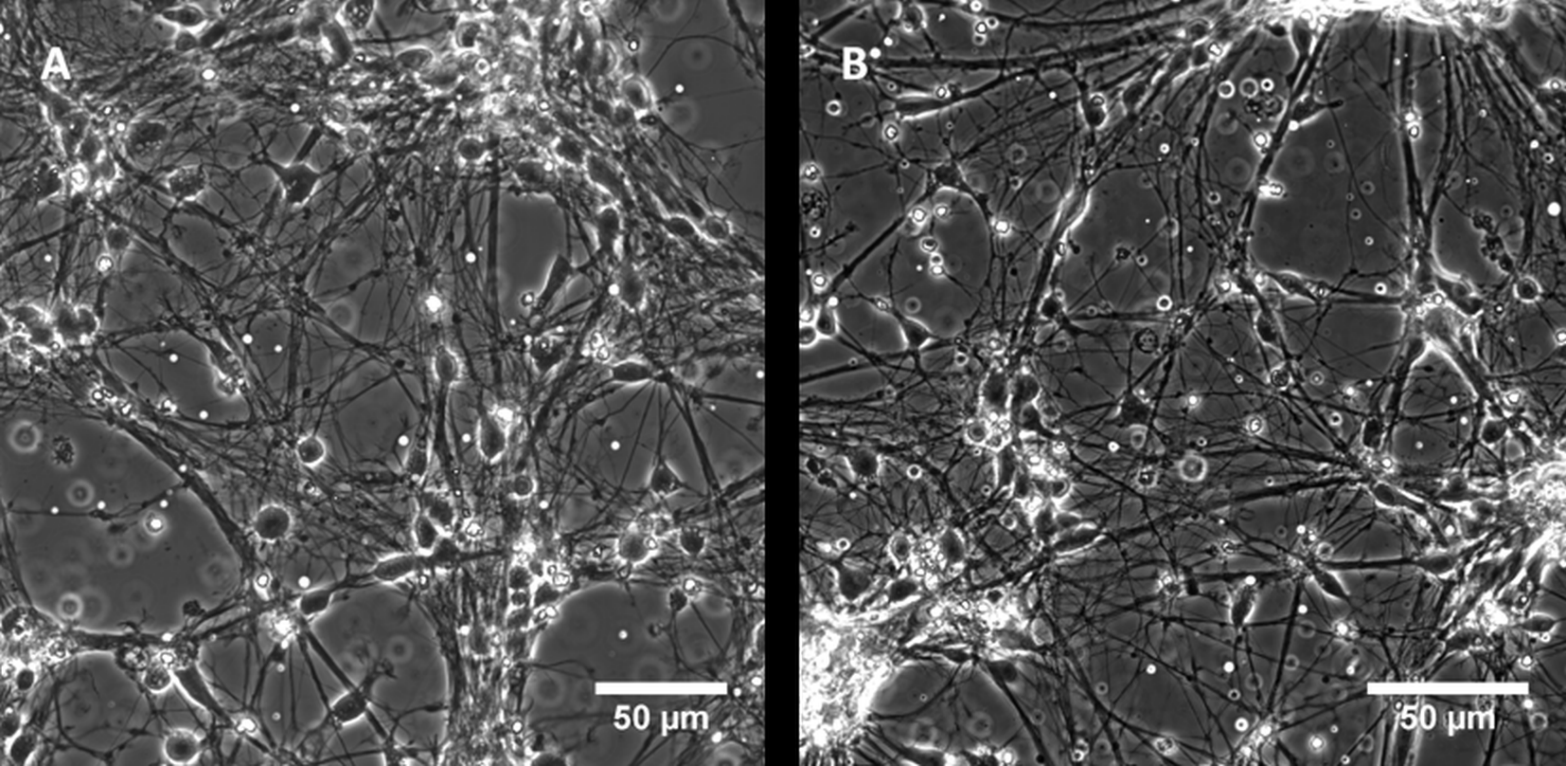
Cortical neurons behave normally when grown on our silicone elastomer substrates. Cortical neurons, grown 5 days on soft PDMS elastomer without inhibitor, system 1, r = 0.71, 0.5 ppm catalyst (A) and with inhibitor, system 3, r = 3.18, 1 ppm catalyst, 2.16% inhibitor (B). Please note that a stoichiometric ratio as high as shown in B) can be achieved only in system 3 due to its long work time.

Developing an elastomer system from few, well characterized basic constituents we were aiming for an ultrasoft material with high biocompatibility. For two reasons this property was explored using primary neurons. On one hand, these cells are well known to survive only at the best possible conditions and, on the other hand, their physiological environment, brain tissue, is extremely soft. Cortical neurons were found to survive well on silicone elastomer substrates with (system 3) and without (system 1) inhibitor for at least 5 days. In all cases they formed many thin and long protrusions, cf. Fig 8. Even after five days usual branching occurred both on system 1 and as well as on system 3, which contains theoretically the most toxic component. No negative influence could be observed; this clearly indicates very good biocompatibility.

## Discussion

The central goal of this work was to generate ultrasoft cell culture substrates for mechanobiology, figuratively speaking “artificial tissues” with mechanical properties similar to native tissues. Starting from commercially available PDMS precursor polymers we followed different approaches toward very soft elastomers described in literature. All substrates were carefully investigated by oscillatory shear rheology to precisely quantify its shear modulus G.

We achieved PDMS elastomers, with a shear modulus tunable over a broad range from G_0_ = 0.3 kPa to G_0_ = 42.6 kPa depending on i) catalyst concentration, ii) stoichiometric ratio r between multi-functional crosslinker and bifunctional network strands (system 1 –neat networks), iii) the amount of added “zero-functional” inert PDMS that swells the network (system 2 –swollen networks), and iv) the density of network defects that can be adjusted by introducing monofunctional network strands (system 3 –inhibited networks). Before we enter a detailed discussion of the possibilities and pitfalls in using these four control parameters to achieve ultrasoft elastomers, we will describe how the measured rheological response can be mathematically described and modeled. This serves a twofold purpose. On one hand, such a mathematical description facilitates systematic comparison to previous work and, on the other hand, it enables the preparation of silicone elastomers with predefined rheological properties.

### Frequency dependence of dynamic modules and data analysis

In their landmark publications Winter and Chambon [24, 31] observed that at the gel point storage and loss module are equal. The characteristic length and time scales of such a critical gel diverge which leads to the typical power law G' = G'' ~ ω^0.5^ valid for all frequencies. As can be clearly seen in Figs 3–6, our systems deviated from this universal behavior. One obvious reason for this is that we investigated weakly crosslinked, but still fully cured samples that were therefore beyond the gel point.

Building on this, Jensen et al. [33] introduced the following model for weakly crosslinked gels,

$$G'\left(\omega \right)=\frac{\pi S\omega^{n}}{2\Gamma \left(n\right)\cos\frac{n\pi }{2}}+G_{0}$$ (3)

$$G''\left(\omega \right)=\frac{\pi S\omega^{n}}{2\Gamma \left(n\right)\sin\frac{n\pi }{2}}$$ (4)

where G_0_ denotes equilibrium shear modulus, S network stiffness, Γ the Gamma function, and n is a network specific exponent defining the power law slope. Unfortunately, this model was not sufficient to describe our data. First, it predicts a ratio between storage and loss module that is far from our observation. Second, it predicts a constant exponent n over the full range of frequencies. However, as can be clearly seen from our data, e.g. Fig 4 and especially Fig 5, this was not the case. Especially the latter observation thwarted the use of the Jensen et al. model for fitting our data.

In a practical approach for parametrizing our data we therefore proposed a new model. We started from the Maxwell model [22], introduced an additional elastic term as in Jensen et al., and, finally, applied a distribution of Maxwell times [34] analogous to the Cole-Davidson [35, 36] or Havriliak-Negami function [37]. This gave the following empirical equations:

$$G'\left(\omega \right)=G_{r}\frac{{\left(\omega \tau_{1}\right)}^{2\alpha }}{{\left(1+{\left(\omega \tau_{1}\right)}^{2\alpha }\right)}^{\beta }}+G_{0}$$ (5)

$$G''\left(\omega \right)=G_{r}\frac{{\left(\omega \tau_{2}\right)}^{2\alpha }}{{\left(1+{\left(\omega \tau_{2}\right)}^{4\alpha }\right)}^{\beta /2}}$$ (6)

Here, *G*_*0*_ is again the equilibrium shear modulus, i.e., the low frequency limit of G' originating from permanent crosslinks, *G*_*r*_ is the rubber plateau modulus resulting at high frequencies from transient crosslinks formed by entanglements, τ is the characteristic Maxwell time that we found to be different for both modules and α, β are the two exponents describing width and asymmetry of the distribution function.

**Fig 9:**
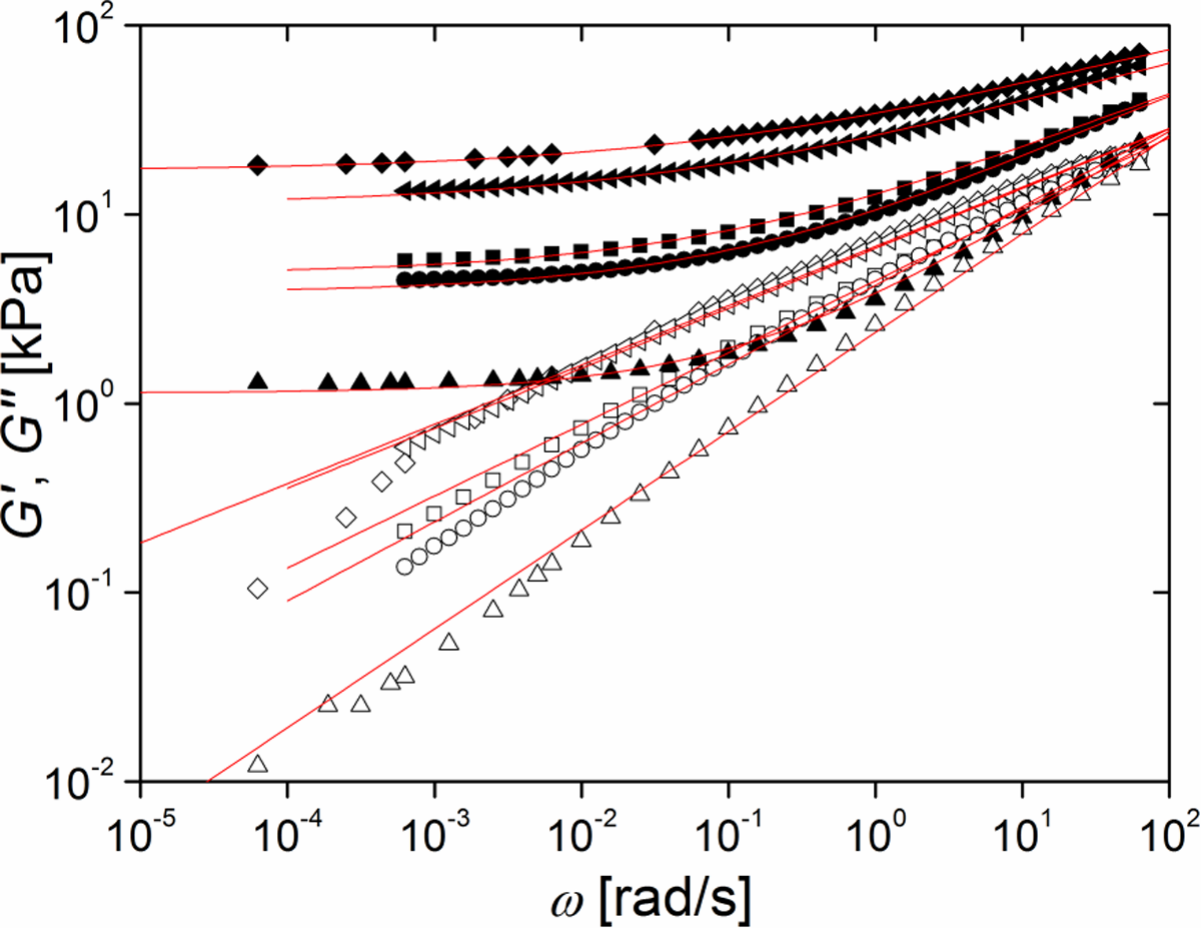
Rheological response of weakly crosslinked elastomers can be described by an empirical model. Fitting of rheological data of neat elastomers (system 1) using the modified Maxwell model, Eqs 5 and 6; frequency dependence of storage (G', solid symbols) and loss modules (G", open symbols); Diamonds: r = 1.28; Triangles, left: r = 1.14; Squares: r = 1.00; Circles: r = 0.92; and Triangles, up: r = 0.71; red lines are fit curves. Raw data can be found in S9 Dataset.

This empirical fitting model quantitatively described our experimental data at all stoichiometric ratios reasonably well (cf. Fig 9). We fixed the parameter β = 1 and G_r_ = 200 kPa to achieve the literature value for the rubber plateau of PDMS at high frequencies [18]. The stretching exponent α slightly decreased with increasing stoichiometric ratio from α = 0.26 to 0.16. We had to use different characteristic times τ for G' and G" with a ratio τ_2_/τ_1_ starting with approximately 0.04 at r = 1.28 and finally approaching 1 with decreasing r.

Please note that our fit model, Eqs 5 and 6, is an entirely empirical model. Most importantly, it violates the Kramers-Kronig relation that can be derived for general linear response functions, i.e. also for G' and G'', with the only assumption of causality [38]. Therefore, our model function must break down in some regimes outside the measurement window. However, such a violation of the Kramers-Kronig relation was already reported by Frankaer et al. [20].

In the remaining sections we focus on how the equilibrium shear modulus G_0_ is tunable by the control parameters catalyst concentration, stoichiometric ratio, filler molecular weight and amount of inhibitor. All four parameters finally influence the number density of elastically effective crosslinks, cf. Eq 1, and will be discussed separately in the following.

### System 1 –neat networks: Effect of the stoichiometric ratio

Variation of the stoichiometric ratio r, see Eq 2, is the standard way to adjust the elasticity of elastomers [20, 24]. For r = 1, that is, stoichiometrically balanced networks, the number of elastically effective crosslinks is maximum since each vinyl group (one at each end of the linear precursor polymers) theoretically finds one hydrosilane group (at the crosslinkers) to react with. For stoichiometrically unbalanced networks, r≠1, either vinyl groups (r < 1) or hydrosilane groups (r > 1) are in excess and only a fraction of the possible crosslinks per volume can be formed. Therefore, stoichiometric balanced networks should exhibit the highest equilibrium shear module with similar decrease for increased or decreased r. In summary, a roughly symmetric curve with a maximum at r = 1 is expected when plotting elastic modulus versus stoichiometric ratio.

In real networks, a broad maximum at slightly larger values at r ≈ 1.3 is found experimentally [18, 20, 25]. Moreover, the elastic modulus decreases rapidly at r<1 and is almost constant at r > 1.3. For commercial PDMS network kits, even larger stoichiometric ratios, r ≈ 2, are proposed to guarantee network formation [25].

Please note that we used a high molecular weight copolymer with a functionality of f_cl_ = 51 as crosslinker instead of the three- or tetra-functional molecular siloxanes used in most publications on silicone elastomers. Although a recent review [39] concludes that most data for high–functionality crosslinkers can be finally mapped onto those for three- or tetra-functional molecular crosslinkers, the high functionality plays a major role. The formation of an elastically effective crosslink requires that at least three polymer chains connect at one point. Therefore, even if only three of the 51 hydrosilane groups of the copolymer reacted with a vinyl group, the whole copolymer crosslinker was elastically effective which enhanced the chances to reach the ultrasoft regime before the infinite network broke down.

Weakly crosslinked networks were produced with stoichiometric ratios between 1.28 and 0.71, see Fig 4. At high stoichiometric ratios we can compare our results with those of Mrozek et al. [25] who give G_0_ = 30 kPa at r = 1.5 which compares quite favorably with our result (20 kPa at r = 1.28). Moreover, our results show the same general trend as those of Frankaer et al. [20]. However, the absolute values of G_0_ reported by these authors differ from our results. At low stoichiometric ratios they found much softer elastomers (0.1 kPa at r = 0.8 compared to our result of 1 kPa at r = 0.71), whereas at higher ones their data and ours agree again better (7.9 kPa at r = 0.95 versus 4.5 kPa at r = 0.93). While we certainly cannot give a definite reason for this discrepancy, we believe that at least parts of it can be explained by the fact that Frankaer et al. had to use time-temperature superposition and extrapolation to obtain the equilibrium shear module whereas it was measured directly in our experiments.

### System 2—swollen networks: Effect of filler molecular weight

In our experiments on swollen networks, the equilibrium shear modulus G_0_ was markedly reduced by addition of 25% inert polymer but showed no systematic dependence on the molecular weight, M_w_, of the filler, see Fig 6. This is in accord with literature, see, for example, Fig 2E in reference [25]. There are three possible reasons for a non monotonic dependence of G_0_ on M_w_: limited miscibility, crossing the critical molecular weight, M_c_, above which dynamic crosslinks are formed or a kinetic effect due to the high molecular weight reaction medium.

Limited miscibility appears unlikely because filler and elastomer are formed from identical chemical building blocks and for mixtures of homopolymers miscibility is always given irrespective of molecular weight [40]. Moreover, none of the typical indicators for demixing, like increased turbidity or oil droplets on gel surfaces, was seen.

Concerning the effect of crossing M_c_, we can exploit the analogy between covalent and transient networks and estimate the expected reduction in equilibrium shear modulus G_0_ upon addition of filler polymers. For entangled melts of linear polymers (transient networks) Rubinstein and Colby [41] predicted a reduction in G(Φ) = G_0_ Φ^7/3^ by swelling with a molecular solvent under theta conditions, i.e. identical interactions between monomer-monomer and monomer-solvent. The same should hold for a good solvent. In our experiments the network volume fraction was kept constant at Φ = 0.75 resulting in an approximately 50% decrease of G_0_. This is comparable to the observed effects at molecular weights of the filler polymer of 68 kg/mol and 139 kg/mol. Surprisingly, for the lowest molecular weight, 28 kg/mol, we found an even higher reduction of about 64%.

Kinetic effects due to filling with an inert polymer are most likely important during the initial phase of crosslinking when only small molecular weight products have formed yet. During this phase the additional macromolecules increase viscosity markedly, resulting in slower kinetics. At later stages, already large and highly branched network fragments, so called “lattice animals” [42], have formed, and the overall viscosity is dominated by these reaction products. Thus, little influence of the filler is expected during these later phases.

Taken together, simple dilution of mechanically active crosslinks combined with some influence of entanglements and reaction kinetics seem sufficient to rationalize the observed dependence of the equilibrium shear module on the molecular weight of filler polymers.

**Fig 10:**
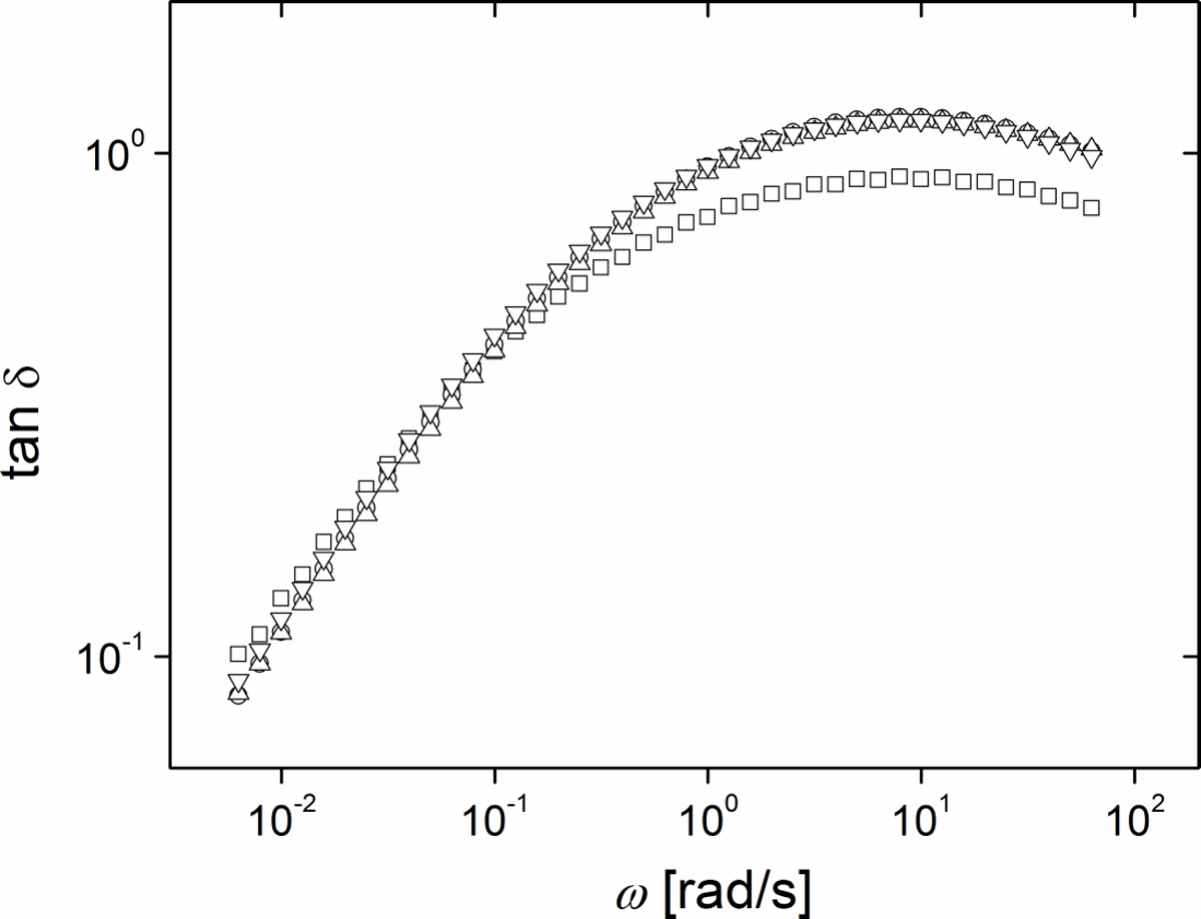
The loss factor tan δ = G''/G' is independent of filler molecular weight. All samples stoichiometric ratio 0.71 and 0.5 ppm catalyst. Squares: Neat elastomer (system 1); all other systems 25% inert silicone polymer added with molecular weights of 28 kg/mol (circles), 68 kg/mol (up triangles), and 139 kg/mol (down triangles), respectively. Raw data can be found in S10 Dataset.

Interestingly, all three swollen elastomers studied exhibited similar loss factors tan **δ** = G''/G', without dependence on molecular weight or frequency, cf. Fig 10. This underlines the relative insignificance of the molecular weight of the inert polymer used for swelling the network.

In essence, adding inert macromolecules to swell the system is a viable approach to lower the elasticity of silicone elastomers. However, in our hands reliability was not as good as we had hoped.

### System 3 –effect of inhibition

The last approach tested was partial inhibition of crosslinking. Here, we added only a small amount, about 0.2–2%, of a low molecular weight compound that reacted with the hydrosilane groups of the crosslinker and therefore inhibited the formation of elastically active network strands. In this approach, we intentionally introduced network defects, so called dangling ends, to lower network elasticity. A related approach was introduced by Cai et al. [18], but these authors started from bottle brush precursor polymers to generate networks with controlled amounts of dangling ends. This approach enabled precise tuning of the final elasticity by adjustment of the molecular weight of the brush, but required sophisticated chemical synthesis. By use of oligomers instead of polymers as dangling ends we circumvented the laborious synthesis step and additionally achieved network elasticities one order of magnitude below those reported by Cai et al. For our system r = 1.28, addition of 2% of inhibitor enabled preparation of elastomers with very low modulus, G_0_ = 0.4 kPa, i.e. well in the ultrasoft regime like brain tissue. Moreover, in contrast to swollen networks (system 2), the mechanical response of even the softest inhibited networks was dominated by elasticity (storage module) at all frequencies. Please note that the pure filler effect is negligible. Addition of such small amount would reduce the equilibirium shear modulus only from 18.3 kPa to 17.5 kPa following Rubinstein and Colby [41].

A practical advantage of the inhibited system is that to produce elastomers with predefined stiffness one can start from the same precursor mixture at fixed stoichiometric ratio r and just vary the amount of inhibitor. By using larger stoichiometric ratios, G_0_ can be tuned within a large range, from ultrasoft to stiffness beyond those observed in physiological conditions (some 100 kPa at most). Another important and not yet addressed point is that the low molecular weight compound can be utilized to introduce functional groups. For example, the monovinyl-terminated alkane compounds used here had an additional bromide group enabling chemical surface modification of the elastomer by ATRP reaction following [43]. Please note, that even though bromide groups were present, all system 3 elastomers gave very good results in cell culture.

From a practical point of view, the most important advantage of system 3 is that curing is inhibited at room temperature enabling long work times and difficult preparations like soft replica molding [44, 45] with these elastomer systems.

### Master curve

**Fig 11:**
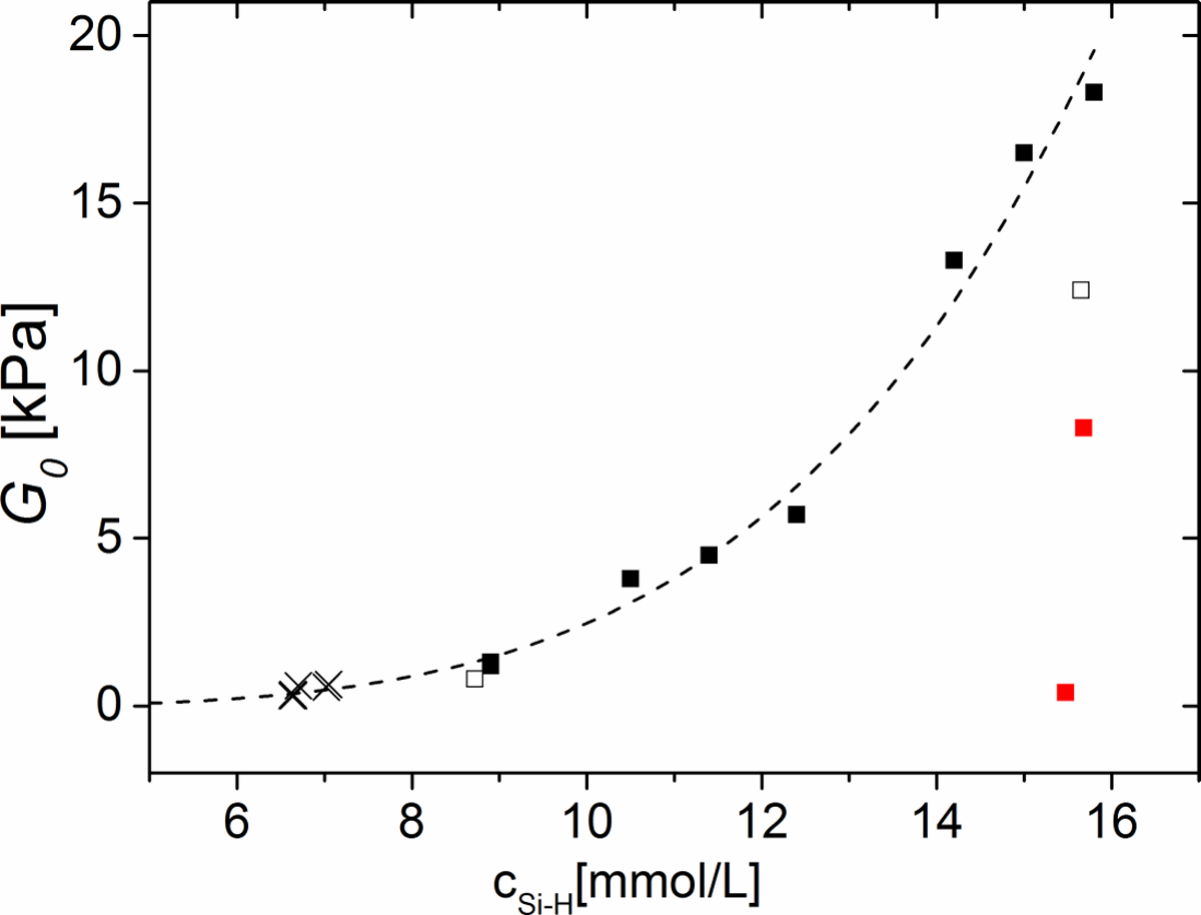
The equilibrium shear module of silicone elastomers is controlled by the concentration of hydrosilane c_Si-H_. Data for PDMS elastomers: Squares, black: system 1, at a catalyst concentration of 0.5 ppm (solid symbols) and less (open symbols); Crosses: system 2; Squares, red: system 3; Dashed line is an empirical fit, Eq 7, cf. text. Numerical values can be found in S11 Dataset.

In Fig 11 we show the measured equilibrium shear module G_0_ to evaluate all important effects of the different approaches, namely i) reduction of the catalyst concentration, ii) variation of the crosslinker stoichiometric ratio r, iii) addition of inert filler polymers of different molecular weight, and, finally, iv) partial blocking of functional groups of the crosslinker. For generating this kind of master curve the number density of elastically effective crosslinks has to be expressed as the concentration of hydrosilane groups.

The data of neat and swollen networks collapsed into one curve, while the data of catalyst and inhibitor concentration significantly dropped below this for corresponding values of c_Si-H_. The curve shows only an increase, but neither a maximum nor a plateau. This is probably due to the relative small excess of crosslinker as described above.

The ascending part of our data can be described by the following empirical equation

$$G_{0}=ac_{Si-H}^{}{}^{n}+b$$ (7)

if c_Si-H_ is given in mmol/L a = 7.8 10^−5^ kPa, b = 0.03 kPa, and n = 4.5. This can be used to predict mechanical properties even in the ultrasoft regime.

## Conclusions

In this project we aimed at elastomer substrates for mechanobiology. These materials must be easy to prepare and use, fully biocompatible, transparent, and exhibit predefined stiffness in the physiologically relevant range from few 100 Pa to some 100 kPa. PDMS based elastomers were used because they can be prepared from few, well defined, and long-lived chemicals. Moreover, these elastomers can be stored for long periods and they are highly biocompatible. Here, we tested different approaches to tune the stiffness of such elastomers as measured by oscillatory rheology. Given G_0_ values of all ultrasoft PDMS elastomers were directly and unambiguously determined in the low frequency range without any need for time-temperature superposition and application of fitting models. In essence, the spatial density of mechanically active crosslinks turned out to be the decisive control parameter in this endeavor. The following approaches were tested to tune elasticity: varying the stoichiometric ratio between crosslinker and polymer, swelling the network with inert polymers, and, finally, inhibition, that is, introducing dangling ends by a monofunctional compound that competes with the multifunctional crosslinker for the active groups on the polymer. All approaches were suitable to vary the equilibrium shear modulus G_0_ of PDMS elastomers. In terms of the ultimate softness reached, inhibition was superior to filling which in turn was superior to sparse crosslinking. Luckily, inhibited networks exhibited additional beneficial properties. Most importantly, they could be easily processed at room temperature because crosslinking starts only at elevated temperatures and they are formed from few and chemically defined starting materials. As additional benefits, they exhibit functional groups for chemical surface modification and their mechanical response is dominated by elasticity over the full frequency range.

To conclude, the presented PDMS elastomers are promising candidates as ultrasoft cell substrates to precisely model the biomechanics of even the softest tissues as brain.

## Supporting information

S1 Table Dependence of work time on preparation conditions. Work times were determined as described in the figure caption of S1 Fig.
(DOCX)

S2 Table Influence of temperature on equilibrium shear module. Equilibrium shear modules G_0_ measured at room temperature, 20°C, and at 37°C. System 1 for all samples. As expected, increasing temperature shifted the equilibrium shear module to higher values. On average we found a 6% increase of equilibrium shear module for this 5.8% increase in absolute temperature. This is in full accord with expectation, Eq 2.
(DOCX)

S1 Fig Work time of curing elastomers can be determined by rheology. Upon cross-linking the material behavior changes from fluid to solid. The time point, *t*_*g*_, of this crossover, also called gel point, is customarily determined from the crossing of *G’* (black solid squares) and *G”* (black open squares) in time sweeps performed at *ω* = 6.28 rad/s during the curing reaction. This corresponds to a loss tangent, tan δ, (red squares) of 1. Because material processing like casting or molding requires predominantly liquid behavior the work time is defined as *t*_*g*_. The shown example was taken at the following conditions: stoichiometric ratio r = 1.14, catalyst concentration *w*_*Pt*_ = 0.50 ppm, frequency *ω* = 6.28 rad/s, strain γ = 1%, and 20°C temperature.
(TIF)

S2 Fig PDMS elastomer mechanical properties depend on temperature. The equilibrium shear module of an ideal elastomer is proportional to temperature, cf. Eq 2. Because experiments on living cells are done at physiological temperature, 37°C, and all rheological experiments reported in the main body of this publication were done at room temperature, 20°C, the influence of this temperature shift on the elastic properties of our PDMS-based elastomers had to be checked. Here shown is the viscoelastic behavior of a PDMS elastomer, stoichiometric ratio r = 0.71, at different temperatures and at fixed strain of 1%. Storage (G', solid symbols) and loss (G'', open symbols) modules measured at 37°C (circles) and 20°C (squares). The inset shows zoom-in on the low frequency behavior of the storage module. Raw data can be found in S12 Dataset.
(TIF)

S3 Fig The precursor polymer is of negligible resistance. To estimate the effect of non-reacted precursor material we also measured the rheological behavior of the high molecular weight vinyl component. The rheological properties of the crosslinker were not measured because its viscosity was about 20 times lower than that of the vinyl terminated polymer. Shown are storage (solid symbols) and loss (open symbols) modules for precursor polymer (stars) and elastomer samples (all system 1) of stoichiometric ratio 1.28 (squares), 1.00 (circles), and 0.71 (triangles); strain 1%. Raw data can be found in S13 Dataset.
(TIF)

S4 Fig Higher molecular weight precursors yield softer elastomers. In system 1, neat elastomers, the number density of crosslinks was mostly determined by the volume of the vinyl-terminated precursor polymer. The underlying reason was as follows: The density of functional groups was much lower in the bivalent, high molecular weight precursor polymer as compared to the multivalent, low molecular weight crosslinker. Thus the precursor made up the bulk of neat elastomer samples. From Eq 2 we therefore expect that the equilibrium shear module of such samples should depend inversely on the molecular weight of the precursor. This was tested by decreasing the molecular weight of the precursor from 155 kg/mol (squares) to 117 kg/mol (circles) and measuring the viscoelastic response of both samples. For the lower molecular weight precursor we found G_0_ = 1.6 kPa while the higher molecular weight substance resulted in 1.2 kPa, exactly as expected. Samples are all system 1 with a stoichiometric ratio of 0.71. Storage (solid symbols) and loss (open symbols) modules measured at 1% strain are plotted. Raw data can be found in S14 Dataset.
(TIF)

S1 Dataset Raw data of Fig 2. (XLSX)

S2 Dataset Raw data of S1 Fig. (XLSX)

S3 Dataset Raw data of Fig 3. (XLSX)

S4 Dataset Raw data of Fig 4. (XLSX)

S5 Dataset Raw data of Fig 5. (XLSX)

S6 Dataset Raw data of Table 2. Sol fraction was determined as described in Materials and Methods. Shown are weights of samples before (m_sample_) and after (m_network_) solvent extraction as well as the weight of the extracted material after drying (m_sol_).
(DOCX)

S7 Dataset Raw data of Fig 6. (XLSX)

S8 Dataset Raw data of Fig 7. (XLSX)

S9 Dataset Raw data of Fig 9. (XLSX)

S10 Dataset Raw data of Fig 10. (XLSX)

S11 Dataset Numerical values of data shown in Fig 11. (XLSX)

S12 Dataset Raw data of S2 Fig. (XLSX)

S13 Dataset Raw data of S3 Fig. (XLSX)

S14 Dataset Raw data of S4 Fig. (XLSX)
