# Supplementary material for: Chemically defined, ultrasoft PDMS elastomers with selectable elasticity for mechanobiology
Source: PLoS One. 2018 Apr 6;13(4):e0195180. doi: 10.1371/journal.pone.0195180 (PMC5889068; doi:10.1371/journal.pone.0195180)
Supplement: S1 Table — Work times were determined as described in the figure caption of S1 Fig. (DOCX) [file pone.0195180.s001.docx]

| r | 1.28 | 1.28 | 1.21 | 1.14 | 1.00 | 0.92 | 0.84 | 0.71 | 0.71 | 0.58 |
| --- | --- | --- | --- | --- | --- | --- | --- | --- | --- | --- |
| w_Pt_ [ppm] | 0.50 | 0.34 | 0.54 | 0.50 | 0.51 | 0.50 | 0.54 | 0.50 | 0.37 | 0.51 |
| t_g_ [min] | 102 | 187 | 106 | 110 | 141 | 150 | 181 | 278 | ∞ | ∞ |
