# Supplementary material for: Chemically defined, ultrasoft PDMS elastomers with selectable elasticity for mechanobiology
Source: PLoS One. 2018 Apr 6;13(4):e0195180. doi: 10.1371/journal.pone.0195180 (PMC5889068; doi:10.1371/journal.pone.0195180)
Supplement: S2 Table — Equilibrium shear modules G0 measured at room temperature, 20°C, and at 37°C. System 1 for all samples. As expected, increasing temperature shifted the equilibrium shear module to higher values. On average we found a 6% increase of equilibrium shear module for this 5.8% increase in absolute temperature. This is in full accord with expectation, Eq 2. (DOCX) [file pone.0195180.s002.docx]

| r | G_0_ at T = 20°C | G_0_ at T = 37°C | ratio |
| --- | --- | --- | --- |
|  | [kPa] | [kPa] |  |
| 1.14 | 13.3 | 14.2 | 1.07 |
| 1.0 | 5.7 | 6.0 | 1.05 |
| 0.92 | 4.5 | 4.8 | 1.07 |
| 0.84 | 3.8 | 3.9 | 1.03 |
| 0.71 | 1.2 | 1.3 | 1.08 |
