# Supplementary figures and images for: Chemically defined, ultrasoft PDMS elastomers with selectable elasticity for mechanobiology
Source: PLoS One. 2018 Apr 6;13(4):e0195180. doi: 10.1371/journal.pone.0195180 (PMC5889068; doi:10.1371/journal.pone.0195180)

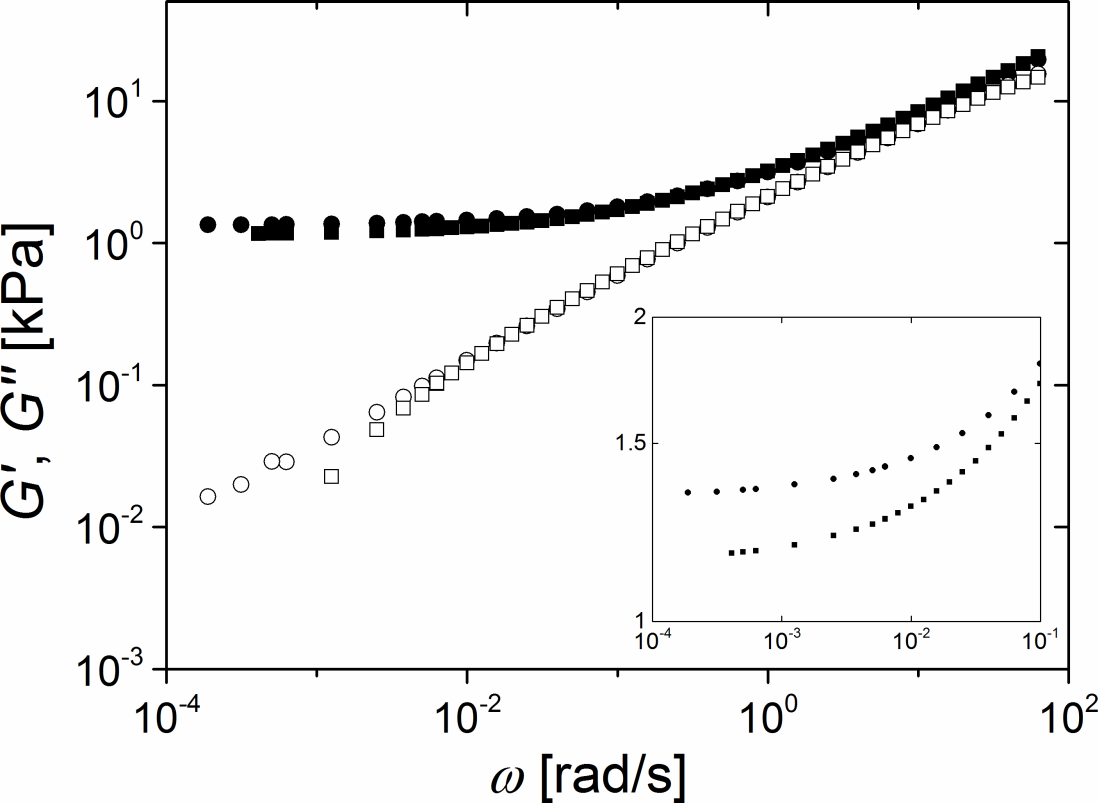

Supplement: S2 Fig — The equilibrium shear module of an ideal elastomer is proportional to temperature, cf. Eq 2. Because experiments on living cells are done at physiological temperature, 37°C, and all rheological experiments reported in the main body of this publication were done at room temperature, 20°C, the influence of this temperature shift on the elastic properties of our PDMS-based elastomers had to be checked. Here shown is the viscoelastic behavior of a PDMS elastomer, stoichiometric ratio r = 0.71, at different temperatures and at fixed strain of 1%. Storage (G', solid symbols) and loss (G'', open symbols) modules measured at 37°C (circles) and 20°C (squares). The inset shows zoom-in on the low frequency behavior of the storage module. Raw data can be found in S12 Dataset. (TIF) [file pone.0195180.s004.tif]

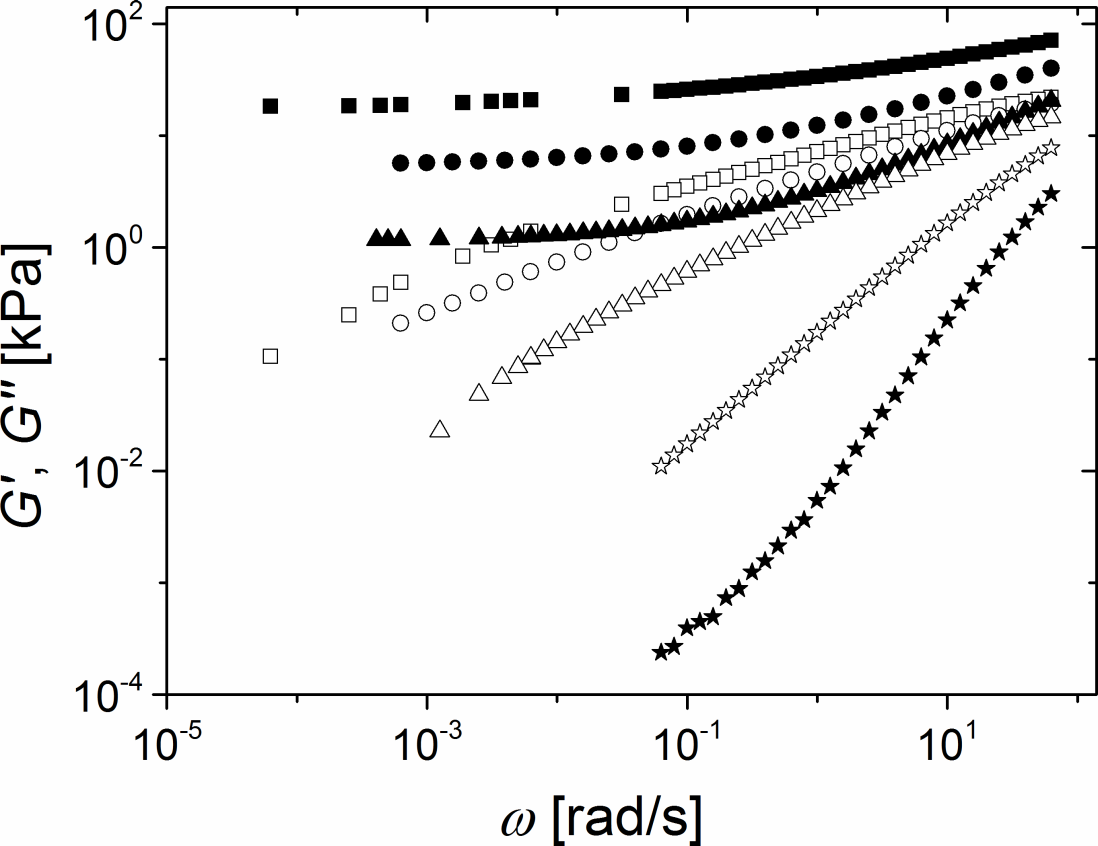

Supplement: S3 Fig — To estimate the effect of non-reacted precursor material we also measured the rheological behavior of the high molecular weight vinyl component. The rheological properties of the crosslinker were not measured because its viscosity was about 20 times lower than that of the vinyl terminated polymer. Shown are storage (solid symbols) and loss (open symbols) modules for precursor polymer (stars) and elastomer samples (all system 1) of stoichiometric ratio 1.28 (squares), 1.00 (circles), and 0.71 (triangles); strain 1%. Raw data can be found in S13 Dataset. (TIF) [file pone.0195180.s005.tif]

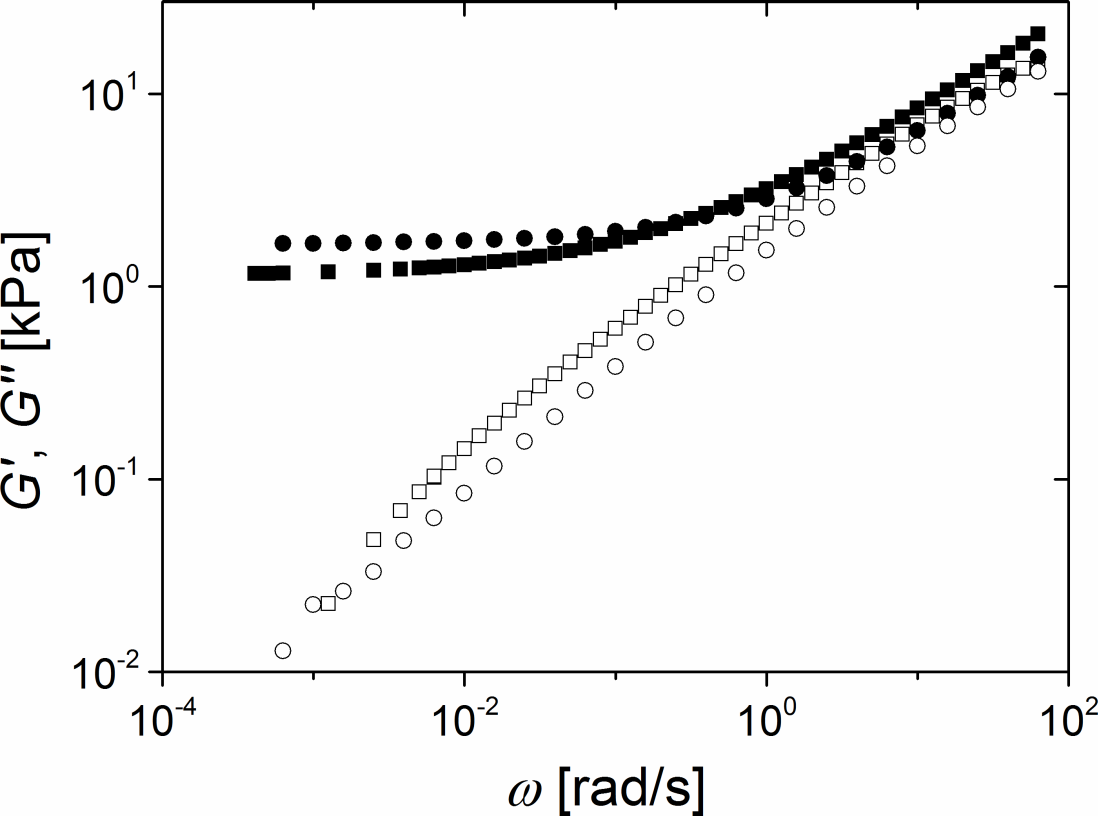

Supplement: S4 Fig — In system 1, neat elastomers, the number density of crosslinks was mostly determined by the volume of the vinyl-terminated precursor polymer. The underlying reason was as follows: The density of functional groups was much lower in the bivalent, high molecular weight precursor polymer as compared to the multivalent, low molecular weight crosslinker. Thus the precursor made up the bulk of neat elastomer samples. From Eq 2 we therefore expect that the equilibrium shear module of such samples should depend inversely on the molecular weight of the precursor. This was tested by decreasing the molecular weight of the precursor from 155 kg/mol (squares) to 117 kg/mol (circles) and measuring the viscoelastic response of both samples. For the lower molecular weight precursor we found G0 = 1.6 kPa while the higher molecular weight substance resulted in 1.2 kPa, exactly as expected. Samples are all system 1 with a stoichiometric ratio of 0.71. Storage (solid symbols) and loss (open symbols) modules measured at 1% strain are plotted. Raw data can be found in S14 Dataset. (TIF) [file pone.0195180.s006.tif]
