# Supplementary material for: Chemically defined, ultrasoft PDMS elastomers with selectable elasticity for mechanobiology
Source: PLoS One. 2018 Apr 6;13(4):e0195180. doi: 10.1371/journal.pone.0195180 (PMC5889068; doi:10.1371/journal.pone.0195180)
Supplement: S6 Dataset — Sol fraction was determined as described in Materials and Methods. Shown are weights of samples before (msample) and after (mnetwork) solvent extraction as well as the weight of the extracted material after drying (msol). (DOCX) [file pone.0195180.s012.docx]

| System 1 | | | | | | | | |
| --- | --- | --- | --- | --- | --- | --- | --- | --- |
| r | 1.28 | | | | 0.84 | | | |
| sample | a | b | c | d | a | b | c | d |
| m_sample_ [mg] | 160 | 149 | 333 | 529 | 245 | 179 | 481 | 417 |
| m_network_ [mg] | 91 | 103 | 231 | 393 | 110 | 84 | 255 | 185 |
| m_sol_ [mg] | 72 | 52 | 104 | 143 | 134 | 94 | 229 | 232 |
| m_network_/m_sample_ [%] | 57 | 69 | 69 | 74 | 45 | 47 | 53 | 44 |
| m_sol_/m_sample_ [%] | 45 | 35 | 31 | 27 | 55 | 53 | 48 | 56 |
| total [%] | 102 | 104 | 101 | 101 | 100 | 99 | 101 | 100 |
| average network [%] | 67 | | | | 47 | | | |
| average solfraction [%] | 35 | | | | 53 | | | |

| System 1, continued | | | | | | | | |
| --- | --- | --- | --- | --- | --- | --- | --- | --- |
| r | 0.71 | | | | | | | |
| sample | a | b | c | d | e | f | g | h |
| m_sample_ [mg] | 127 | 215 | 490 | 201 | 194 | 156 | 173 | 254 |
| m_network_ [mg] | 47 | 70 | 191 | 53 | 54 | 43 | 50 | 97 |
| m_sol_ [mg] | 100 | 143 | 299 | 151 | 145 | 139 | 128 | 160 |
| m_network_/m_sample_ [%] | 37 | 33 | 39 | 26 | 28 | 28 | 29 | 38 |
| m_sol_/m_sample_ [%] | 79 | 67 | 61 | 75 | 75 | 89 | 74 | 63 |
| total [%] | 116 | 99 | 100 | 101 | 103 | 117 | 103 | 101 |
| average network [%] | 32 | | | | | | | |
| average solfraction [%] | 73 | | | | | | | |

| System 2, 25% inert PDMS | | | | |
| --- | --- | --- | --- | --- |
| r | 0.71 | | | |
| sample | a | b | c | d |
| m_sample_ [mg] | 185 | 171 | 211 | 342 |
| m_network_ [mg] | 55 | 51 | 35 | 105 |
| m_sol_ [mg] | 135 | 145 | 108 | 231 |
| m_network_/m_sample_ [%] | 30 | 30 | 17 | 31 |
| m_sol_/m_sample_ [%] | 73 | 85 | 85 | 68 |
| total [%] | 103 | 115 | 102 | 98 |
| average network | 27 | | | |
| average solfraction | 78 | | | |
